# Miscellany

**Published:** 1895-10

**Authors:** 


### Miscellany.

An army medical board will be in session at Washington City, D. C., during October, 1895, for the examination of candidates for appointment to the medical corps of the United States army, to fill existing vacancies. Persons desiring to present themselves for examination by the board will make application to the secretary of war, before October 8th, for the necessary invitation, giving the date and place of birth, the place and state of permanent residence, the fact of American citizenship, the name of the medical college from which they were graduated, and a record of service in hospital, if any, from the authorities thereof. The application should be accompanied by certificates based on personal acquaintance, from at least two reputable persons, as to his citizenship, character and habits. The candidate must be between twenty-two and twenty-nine years of age and a graduate from a regular medical college, as evidence of which his diploma must be submitted to the board. Further information regarding the examinations may be obtained by addressing Surgeon-General U. S. Army, Washington, D. C.

